# Development of a Biosensory Computer Application to Assess Physiological and Emotional Responses from Sensory Panelists

**DOI:** 10.3390/s18092958

**Published:** 2018-09-05

**Authors:** Sigfredo Fuentes, Claudia Gonzalez Viejo, Damir D. Torrico, Frank R. Dunshea

**Affiliations:** Faculty of Veterinary and Agricultural Sciences, University of Melbourne, Parkville, VIC 3010, Australia; cgonzalez2@unimelb.edu.au (C.G.V.); damir.torrico@unimelb.edu.au (D.D.T.); fdunshea@unimelb.edu.au (F.R.D.)

**Keywords:** autonomic nervous system, computer vision algorithms, integrated camera system, nonintrusive biometrics, sensory evaluation

## Abstract

In sensory evaluation, there have been many attempts to obtain responses from the autonomic nervous system (ANS) by analyzing heart rate, body temperature, and facial expressions. However, the methods involved tend to be intrusive, which interfere with the consumers’ responses as they are more aware of the measurements. Furthermore, the existing methods to measure different ANS responses are not synchronized among them as they are measured independently. This paper discusses the development of an integrated camera system paired with an Android PC application to assess sensory evaluation and biometric responses simultaneously in the Cloud, such as heart rate, blood pressure, facial expressions, and skin-temperature changes using video and thermal images acquired by the integrated system and analyzed through computer vision algorithms written in Matlab^®^, and FaceReader^TM^. All results can be analyzed through customized codes for multivariate data analysis, based on principal component analysis and cluster analysis. Data collected can be also used for machine-learning modeling based on biometrics as inputs and self-reported data as targets. Based on previous studies using this integrated camera and analysis system, it has shown to be a reliable, accurate, and convenient technique to complement the traditional sensory analysis of both food and nonfood products to obtain more information from consumers and/or trained panelists.

## 1. Introduction

Sensory is the science applied to acquire, measure, analyze, and interpret the consumer, trained panelist, and/or expert responses to different food and beverages or any other nonfood products experienced through one or more of the five human senses [1]. Taditional methods to assess sensory attributes of a product for any type of test involve the use of paper ballots for self-reported questionaires; however, these are time-consuming in terms of data collection, digitalization, and analysis [2]. Furthermore, other disadvantages can be related to food, beverages, or some nonfood products combined with the use of paper forms, which tend to be un-neat and unclean due to spills and food marks, making the process more difficult to read when collecting data. Therefore, nowadays many companies have developed computer-based software to ease data collection and analysis such as Compusense (Compusense Inc., Guelph, ON, Canada), SIMS 2000 software (SIMS Sensory Software, Morristown, NJ, USA), RedJade sensory software (Curion, Redwood City, CA, USA), and FIZZ sensory software (Biosystemes, Couternon, France), however, these can be cost-prohibitive for many small and medium food and nonfood companies and/or research centers. Furthermore, the aforementioned software only offers the capability of making surveys to gather conscious responses based on traditional sensory tests, but do not incorporate any biometric responses.

Sensory evaluation is divided into three main types of tests: (i) discriminative, (ii) acceptability, and (iii) descriptive. The use of acceptability tests tends to give subjective responses; therefore, there is a need for a larger number of consumers to evaluate the samples to get more consistent results, which is more time-consuming [3,4]. On the other hand, discriminative and descriptive tests are usually conducted using a smaller group of trained panelists and data are more objective. However, the use of continuous scales in descriptive tests, such as a 10 or 15 cm nonstructured scale, through paper ballots makes their data collection more time-consuming as the use of a ruler to measure each response is involved [4,5]. Hence, an automated system to gather results is of high importance in all sensory tests to reduce time and the use of consumables, such as paper and pens.

More recently, sensory studies with consumers have incorporated biometrics, which involve the use of personal identification techniques to obtain physiological data, which are a response to stimuli from the autonomic nervous system (ANS) such as heart rate, body temperature, and facial-expression changes, which help to assess the unconscious responses of participants to different stimuli such as videos, images, real-life situations [6,7], and food and beverages such as chocolate [8] and beer [3,7], respectively. De Wijk et al. [9,10] have conducted studies involving the use of intrusive electrodes to measure the heart rate on the ear lobe, and skin conductance and temperature sensors positioned on fingers. Other authors such as Beyts et al. [11] have measured heart rate using an electrocardiogram with electrodes attached to the participants, and skin temperature using a thermisor under the forearm, while Verastegui-Tena et al. [12] used sensor pads attached to the participants to measure heart rate and electrodermal-transducer sensors for skin conductance. Taamneh et al. [13,14] presented a web-based tool, SubjectBook, to collect, arganize, and visualize multimodal data. However, this tool acts more as a dashboard and, as the aforementioned studies, the SubjectBook tool works by accruing data from wearable sensors to measure physiological responses. The use of contact sensors to measure parameters from the autonomic nervous system such as heart rate and blood pressure, has showed to have an effect on the participants physiological responses [15], as the awareness of being monitored increases their anxiety, increasing their arousal and, thus, modifying their ANS responses. Increasing the anxiety of participants when aware of being monitored also has an effect on their emotional responses as it increases fearfulness, concern, and nervousness [16]. Pavlidis et al. [17] developed a technique to measure breath rate, blood flow, and heart rate using thermal images. On the other hand, authors such as Danner et al. [18] have studied facial expressions using a low-resolution webcam. It is important to note that these studies do not involve an integrated system, as every component is measured independently. This means that the sensory form, video recording, and electrodes for temperature and heart rate have independent hardware and software components, which makes the analysis more difficult. Moreover, the video recording is done during the whole sensory session, which involves the use of more memory space and makes the analysis more difficult as, when just some parts of the session are required, there is a need for manually cutting the videos for the specific times of interest when the stimuli were presented to panellists.

This paper discusses an automatic and nonintrusive method based on the development of an integrated camera system to record videos and thermal images coupled with a biosensory application (app) for Android (Google, Mountain View, CA, USA) Tablet PCs. The biosensory app is able to create digitally customized sensory forms for any type of scale used in sensory tests and can be used remotely with any internet connection. The analyses of videos and images using computer vision algorithms written in Matlab^®^ ver. R2018a (Mathworks Inc., Matick, MA, USA) and FaceReader^TM^ 7.1 software (Noldus Information Technology, Wageningen, The Netherlands) are also explained and discussed. The functionality and performance of the integrated system are presented, along with examples of the outcomes analyzed through principal component analysis obtained using Matlab^®^ ver. 2018a. The biosensory app and the integrated camera system have been already applied for the assessment of different food and beverage products as well as nonfood products such as labels and images using the senroy laboratory booths belonging to the Faculty of Veterinary and Agricultural Sciences (FVAS) from The University of Melbourne (UoM).

## 2. Materials and Methods

### 2.1. Integrated Video- and Thermal-Camera Description

**Figure 1:**
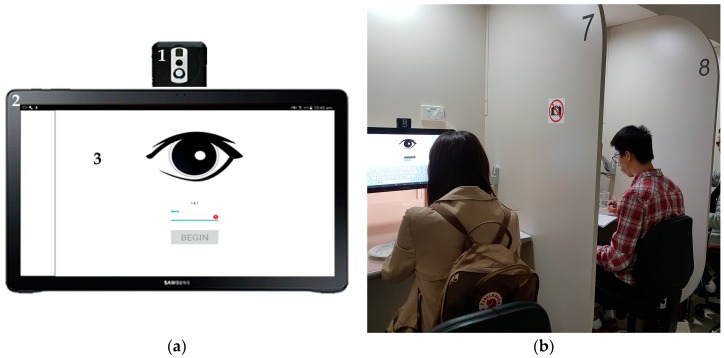
Images of the (**a**) integrated camera system with: (1) the thermal camera FLIR AX8™, and (2) Android tablet displaying the (3) biosensory app start screen; and (**b**) booths in the of the sensory laboratory of the Faculty of Veterinary and Agricultural Sciences of The University of Melbourne, Australia.

An integrated camera system (Figure 1a), which is capable of recording videos and, optionally, capturing infrared thermal images (IRTI) every two seconds was developed. The videos are configured to a resolution of 1640 × 1232 pixels, aspect ratio of 4:3, 30 frames per second (fps), and full field of view (FoV) using the internal camera included in the Android tablet. For the optional use of the thermal camera, the IRTIs were recorded using a FLIR AX8™ camera (FLIR Systems, Wilsonville, OR, USA), which is able to take both thermal and visible images. When using both cameras, these were integrated and connected to a small Raspberry Pi board (Raspberry Pi Foundation, Cambridge, UK) placed at the back of the device, where they were able to connect through a dedicated WiFi network, so they could be automatically controlled by the biosensory app. The integrated cameras were located in each of the 20 booths of the sensory laboratory from FVAS-UoM (Figure 1b). The position of the tablets was adjustable according to the height of each participant.

### 2.2. Integrated System and Biosensory App Development

#### 2.2.1. Biosensory App Development

The biosensory app was developed for Android devices (Google, Mountain View, CA, USA) using Android Studio (Google, Mountain View, CA, USA). It is intended to be used in tablets with Android 5.1 or 6.0, 2 GB random access memory (RAM), a 1920 × 1080 screen, front webcam, and a minimum of 8 GB free space in a Secure Digital (SD) card. The app was designed according to the participants' needs to be easy to use by being self-explanatory and dynamic. Furthermore, it was designed to be easy and flexible for test makers (users) to create new sensory forms and questionnaires according to the type of test and product(s) to evaluate. The sensory forms were designed through a user-friendly configuration file generator in an application program interface (API) on the Amazon Web Service Elastic Compute Cloud (AWS EC2) in which the user is able to add the title of the assessment, codes for samples, select whether or not to randomize the order of samples presentation, and whether or not to repeat the same questions for all samples. A ranking option is also available and can be switched on and off according to the tests’ objective. This feature is flexible as it can be specified in which part of the test the ranking should appear and how many samples need to be assessed for this test. Additionally, the user can define the type of scale to use (categorical scale, face scale, continuous-line scale), specify the descriptors and categories or levels, and if the question is conditional, which means that, depending on the answers given, it will determine which the following question is. It also has the option to add images, videos, and/or sound within the questionnaire to increase the number and type of stimuli to panellists. The API on the AWS EC2 is able to build a configuration file (config file) in JavaScript Object Notation (Json) format, which is then deployed to the tablets to be used for a test.

The biosensory app is also capable of recording videos and, optionally, thermal images while the participant is evaluating the sample and answering the questions only or for the entire session. These videos and images would allow the sensory-session leader to further analyze the panelists’ reactions when tasting the products by assessing heart rate, blood pressure, skin temperature, and facial expressions (explained later). The user is able to use either the integrated system or just the video camera if the thermal camera is not present; this gives the option to use any tablet available as it has the capability of being mobile and work with any internet connection. Furthermore, there is an option of either recording the whole session or selecting the questions and samples in which videos and images are required. The user is also able to select the time to display each question, which allows to determine the video time in case there is a minimum required.

#### 2.2.2. Monitoring-System and Cloud-System Description

**Figure 2:**
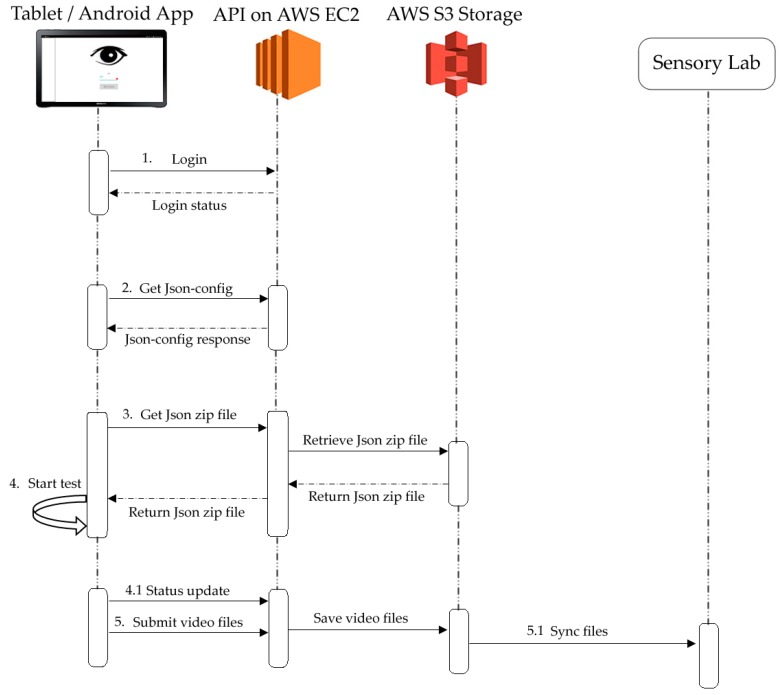
Diagram showing how the integrated system works through the Cloud. Abbreviations: AWS = Amazon Web Service, API = Application Program Interface, EC2 = Elastic Compute Cloud, Json = JavaScript Object Notation.

The integrated system works through a Cloud system that allows it to be used remotely (not only at the FVAS-UoM sensory laboratory) (Figure 2) After downloading and installing the app, the user must (1) log in with a unique username and password, which would aid with the security measures required for data management. Furthermore, the user defines the questions and sample codes in the (2) Json file generator in the API on the AWS EC2, which then is (3) saved into a zip folder and retrieved by the app along with any other files such as video, audio, and/or image to be used for the test; once this is completed, the participants are able to (4) start the test. While the app is being used, it sends (4.1) status updates to the AWS EC2 so that both the user and administrator can monitor remotely which devices are being used, who is taking the test, the system status, and which the next sample to evaluate is. The system is also able to (5) submit and encrypt the files (videos and answers) in real time to the AWS S3 storage, from which the administrator can (5.1) retrieve all decrypted data for further analysis.

### 2.3. How the Biosensory App Works

**Figure 3:**
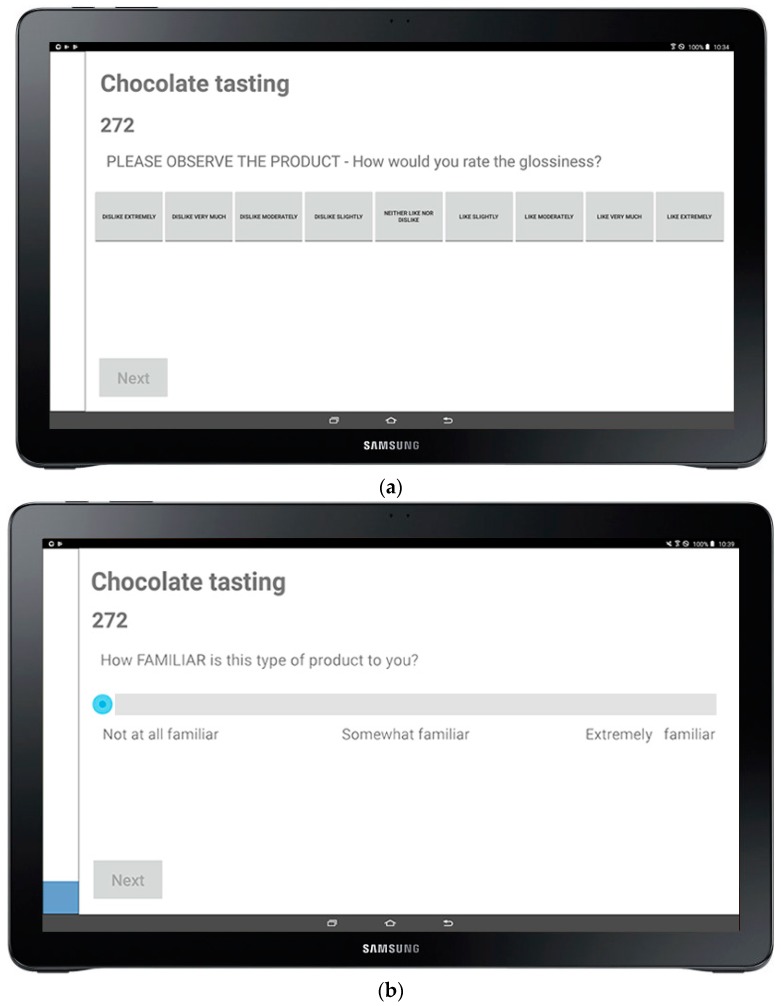
Sensory form as presented in the biosensory app for each sample where: (**a**) shows the categorical scales, and (**b**) the 15-point continuous scale.

Once the Json file is created, it must be sent remotely through the AWS EC2 to all devices (20 devices in the FVAS-UoM sensory-lab case), so that the app can display it. After the file is set up, the participants are required to enter their name and press the “Begin” button (Figure 1). The app then takes the participant to complete the demographics section (age, gender, country of origin, among others), followed by the questionnaire with the pre-established set of descriptors for each sample. In Figure 3, examples of a categorical (Figure 3a) and 15-point nonstructured continuous scales (Figure 3b) are shown.

**Figure 4:**
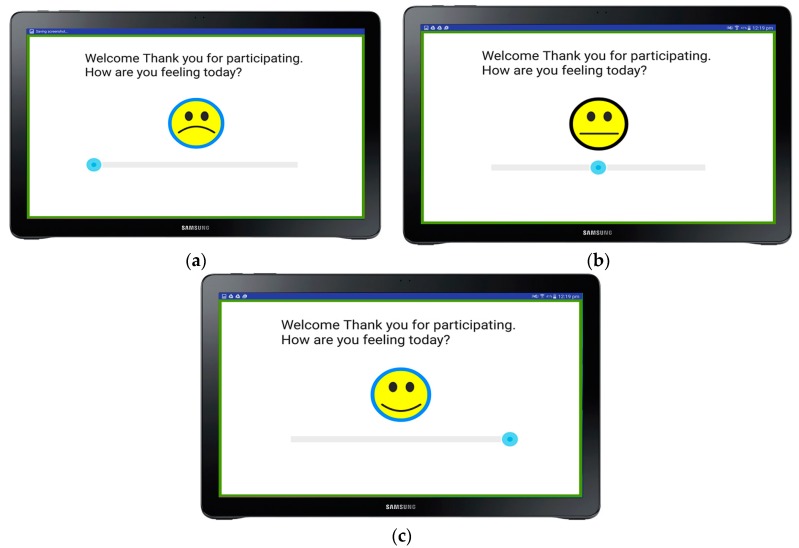
Welcome screen to validate the participants feelings when starting the test, showing: (**a**) the negative or sad face as the lower anchor, (**b**) the neutral for the middle anchor, and (**c**) the happy or positive side of the scale.

The biosensory app has the ability to use a face countinuous scale that goes from very sad (Figure 4a) to very happy (Figure 4b), being the middle a neutral response (Figure 4c). To answer the question, the panelist is required to move the blue circle to the desired position and press the face once it is ready to continue. This can be added as a welcoming screen and will help the user to have a baseline of the participants feeling when starting the test to be able to assess how this is affected along the session.

Once the participant has finished evaluating one sample, a break screen can be added. This allows the participant to take a break if needed and take a palate cleanser during the session. The app will not record videos while this message is shown, thereby providing more stability to the system and reducing the length of videos, which, at the same time, eases the analysis when the camera is set to record the entire session.

**Figure 5:**
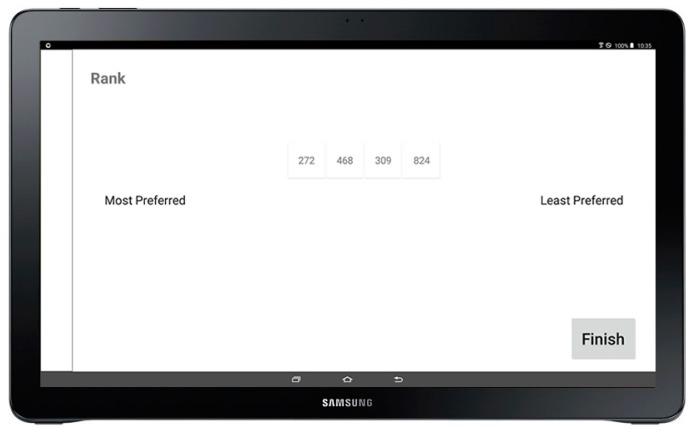
Screen showing the ranking test used to assess preference, ordering samples from most to least preferred.

When the participant has finished evaluating either a set of samples or the whole session (depending on the setting specified), for consumer evaluation, a ranking test to assess preference can be added. As shown in Figure 5, the screen shows the three-digit random codes of each sample and the participant is able to drag and drop the boxes containing the codes to order them from most to least preferred.

When the session is finished, the app saves the results (data file), videos, and images in both the Cloud AWS S3 and internal computer to create backups of the data. The videos are created in Motion Pictures Expert Group-4 (.mp4) format, images in Joint Photographic Experts Group format (.JPG), and the data in a text file (.txt), each saved with the date, time, participants' name, sample, and question number in a folder created for each participant, sample, and question. The text file can be opened using Excel, where data are presented in a pre-established ordered manner (Table 1) to facilitate gathering the values of all participants into a single file. The app is also able to record the specific time at which the participant responded to each question.

### 2.4. Algorithms Used for Image, Video, and Infrared Thermography Analyses

The images recorded during the session are analyzed for facial expressions using FaceReader^TM^ 7.1 software (Noldus Information Technology, Wageningen, The Netherlands). The software uses face-detection algorithms and is capable of detecting movements from different parts of the face, which are related to a database integrated in the program to associate them with distinct expressions. Once these are detected, they are classified into eight different parameters: (i) happy, (ii) neutral, (iii) sad, (iv) scared, (v) surprised, (vi) angry, (vii) contempt, and (viii) disgusted; additionally it is able to calculate two dimentions: (ix) valence and (x) arousal, which are related to the positive and negative responses to the stimuli presented to the participant. Furthermore, it is able to assess the head orientation in the *x*- (*X*-Head), *y*- (*Y*-Head), and *z*- (*Z*-Head) axes.

The videos are then processed to assess the heart rate (HR) and blood pressure (systolic = SP and diastolic = DP) of the participants during the presentation of the stimuli. To obtain these data, videos are processed using an algorithm written in Matlab^®^ ver. R2018a (Mathworks Inc., Matick, MA, USA) based on luminosity changes of the face in the green color component using the photoplethysmography principle. Once the videos are processed using the aforementioned algorithm, a machine-learning model is fed with the input data obtained to calculate the real mean values for heart rate and blood pressure (SP and DP) [19].

Both the thermal and visible images from the infrared camera are separated and the raw data file is obtained using the FLIR^®^ Tools software (FLIR Systems, Wilsonville, OR, USA), to be further analyzed using a code written in Matlab^®^ ver. R2018a (Mathworks Inc., Matick, MA, USA), which is based on the cascade object detector to allow the automatic recognition of the eye section of the participants using the visible image [20]. The code is designed to recognize the entire face in case it is not able to find the area of the eyes. Once it detects the area of interest, the images are coregistered to obtain the maximum temperature in each thermal image, which gives the skin temperature (IR) of the participants [3,7,8].

### 2.5. Examples of the Use of the Integrated Camera System in Sensory Sessions Using Beer and Images as Stimuli

To show a possible application of the integrated camera system and biosensory app, data from *n* = 30 consumers who participated in a sensory session using beer as stimulus is presented. In this session, nine commercial beer samples from different styles (porter, aged ale, kolsh, lager, American lager, pilsner, lambic cassis, lambic framboise, and lambic kriek) were used. Participants were seated in individual booths in the sensory laboratory of FVAS-UoM, Australia. Each individual booth was equipped with an integrated camera system (Figure 1b) and uniform lighting conditions. Participants were asked to taste the sample and respond to the questionnaire presented in the tablet; videos and IRTIs were recorded while participants were tasting the samples. The questions asked of the participants were: (i) foam-staibility liking (FStability), (ii) foam-height liking (FHeight), (iii) aroma liking (Aroma), (iv) bitterness-taste liking (TBitter), (v) carbonation-mouthfeel liking (MCarb), (vi) flavor liking (Flavor), and (vii) overall liking (Overall). Subconscious responses such as the aforementioned eight emotions, two dimensions, head orientation in *x*, *y* and *z*, gaze direction from FaceReader™, plus heart rate and temperature were obtained. All data were analyzed through multivariate data analysis based on principal component analysis (PCA) using Matlab^®^ ver. R2018a. This analysis allows to find relationships between the conscious and subconscious responses when tasting different beer samples. Data of the heart rate and skin temperature changes from two participants (Asian and Westerner) when tasting the porter beer were developed over time to show an example of the results obtained using the computer vision algorithms.

Another sensory session using nine images (three positive, three neutral, and three negative) from the Geneva Affective PicturE Database (GAPED) as stimuli [21] was conducted. This session involved *n* = 59 participants using individual booths in the sensory laboratory of the FVAS-UoM, Australia. As in the previous example, each individual booth was equipped with an integrated camera system (Figure 1b) and uniform lighting conditions. Participants were asked to look at each image and respond to the questionnaire presented in the tablet; videos and IRTIs were recorded while participants were assessing the stimuli. The participants were asked to look at a blank screen for three seconds to neutralize emotions, followed by a seven-second image display; videos were recorded during this task. Following the latter, participants were asked to respond to a face-scale question to assess how the image made them feel. In this example the videos and images were analyzed only for emotions, skin temperature, and heart rate. Multivariate data analysis based on PCA and cluster analysis were performed using Matlab^®^ ver. R2018a. This analysis allows to find relationships between multivariate data and how stimuli are clusterd according to consumers' responses.

## 3. Results from Examples

### 3.1. Results Obtained from the Biosensory App Using the Integrated Camera System for the Sensory Sessions Using Beer Samples as Stimuli

**Figure 6:**
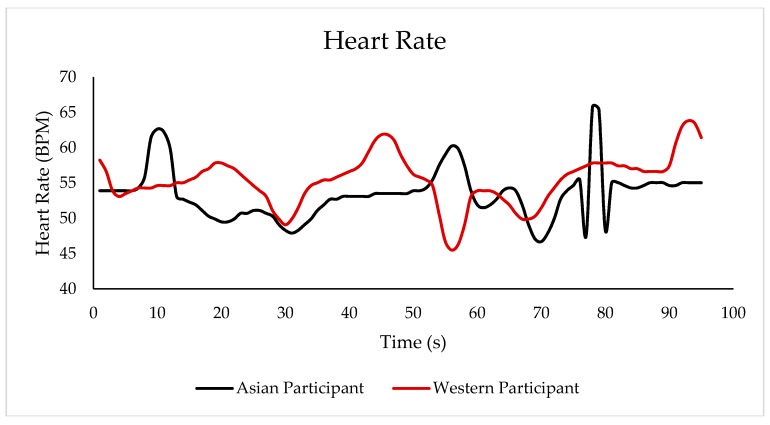
Example of the heart-rate changes over time when tasting a porter beer using the responses of an Asian and a Western participant.

**Figure 7:**
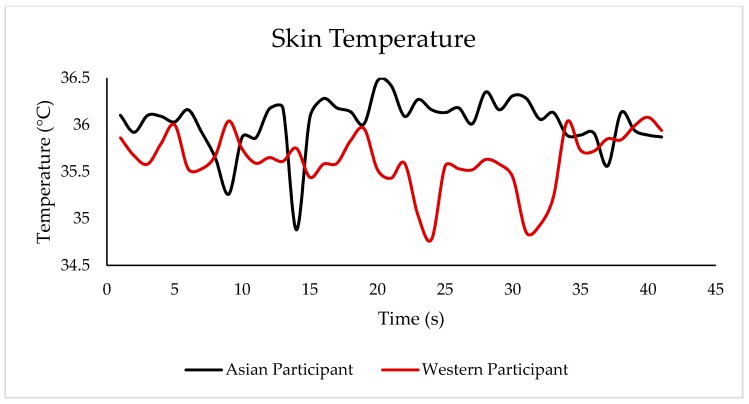
Example of the skin-temperature changes overtime when tasting a porter beer using the responses of an Asian and a Western participant.

Figure 6 shows an example of the heart-rate response of two participants, an Asian and a Westerner, when tasting the porter beer within the time they took to taste the sample. Likewise, Figure 7 shows the results of skin-temperature changes when tasting the same porter sample. In both graphs, it is shown that both participants had a different response when tasting the same sample.

**Figure 8:**
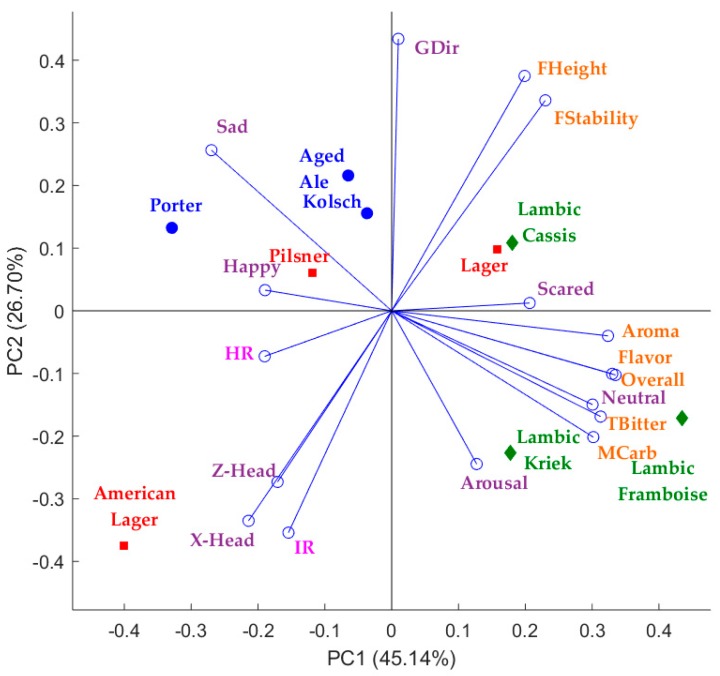
Multivariate data analysis showing the principal components analysis where: IR = body temperature, HR = heart rate and head orientation in *x*- (*X*-Head), *y*- (*Y*-Head) and *z*- (*Z*-Head) axes.

Figure 8 shows the PCA from the beer-tasting test in which the first principal component (PC1) represents 45.14% of data variability, while the second principal component (PC2) accounts for 26.70%, explaining a total of 71.84%. PC1 was mainly represented by conscious responses such as Aroma, Flavour, and Overall liking on the positive side, and sad and *X*-Head on the negative side. On the other hand, PC2 was mainly represented by GDir and FHeight on the positive, and IR and *X*-Head on the negative side of the axis. In this example it can be observed that the IR was negatively related with FHeight and FStability, and positively related with *Z*-Head and *X*-Head. Furthermore, HR had a positive relationship with happy, and a negative relationship with scared and FHeight and FStability. All conscious responses related to taste, aroma, and mouthfeel were positively related with neutral, and negatively related with sad.

### 3.2. Results Obtained from the Biosensory App Using the Integrated Camera System for the Sensory Sessions Using Images as Stimuli

**Figure 9:**
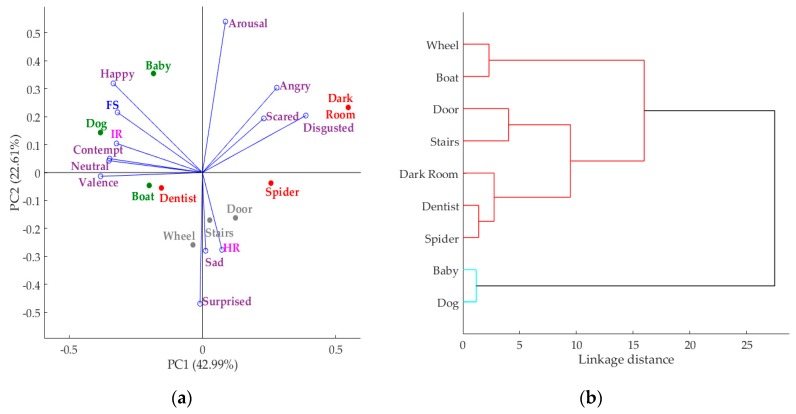
Multivariate data analysis showing: (**a**) principal components analysis where: IR = body temperature, HR = heart rate, and FS = face scale; and (**b**) cluster analysis where *x*-axis = linkage distance and *y*-axis = images.

Figure 9 shows an example of the results obtained from the multivatriate data analysis using the data from GAPED images as part of the biosensory app. In Figure 9a, the PCA is presented and it can be observed that it explains 65.60% of the total data variability (PC1 = 42.99%; PC2 = 22.61%). PC1 was mainly represented by Disgusted and Angry on the positive side, and the emotions Neutral and Contempt as well as Valence on the negative side. PC2 is mainly represented by Arousal on the positive side and Surprised, Sad, and HR on the negative side of the axis. In this figure, the relationship between the negative facial expressions (angry, scared, and disgusted) assessed using FaceReader™, along with their negative relationship with the face scale (FS) used in the test and the positioning of the negative images, can be depicted. Likewise, Figure 9b shows the clusters obtained using the eucledian linkage based on the PCA, in which negative images are found within the same group. Furthermore, it can be seen that positive images such as baby and dog were the highest in happy and the FS, as well as skin temperature. A positive relationship between HR and sad, and between skin temperature and FS and happy, can also be observed.

## 4. Discussions

### 4.1. Application of the Biosensory App Using the Integrated Camera System for the Sensory Sessions Using Beer and Images as Stimuli

Results from both the beer tasting and the presentation of image stimuli showed that the integrated camera system and biometric analysis are an effective way to obtain the conscious and subconscious (physiological) responses from consumers and that these two types of responses are related. Therefore, it is easy to identify the performance of the physiological responses when participants are subjected to different types of stimuli. Some interesting findings in the presented results were the negative relationship between skin temperature and the liking of foam height and stability, and the negative relationship between HR and the scared emotion when tasting beer. Other interesting findings were the positive relationship between heart rate and emotions such as sad and surprised, and between skin temperature and the conscious response from the FS and happy emotion when looking at image stimuli. Some other studies using the presented system and biometric algorithms have obtained useful results, such as the negative relationship between HR and the bitter-taste intensity using a just-about-right (JAR) scale in beer samples [3], the positive correlation between HR and the perceived quality of beer and the positive correlation between happy and liking of foam height when assessing the pouring of the beer samples [7]. Figure 6 and Figure 7 show the responses of heart rate and skin temperature over time while tasting a sample; this continuous analysis can potentially be used for temporal dominance of sensations (TDS) and time-intensity tests. Hence, this integrated system is a useful tool for sensory evaluation to make it easier for participants to use the questionaires, reduce time of analysis, and gather physiological responses remotely, as it has been found that the awareness of being in contact with sensors affects participants' reactions [16]. This system also allows for its mobile use, which would allow to gather data from any part of the world and in any environment. Furthermore, it is capable of being coupled with other biometrics such as eye-tracking and electroencephalogram (EEG) headsets [3,7].

### 4.2. Example of Application of the Biosensory App Using an Integrated Camera System to Other Products to Obtain Machine-Learning Models

This integrated camera system has been used in sensory-evaluation studies of food, beverages, and nonfood products such as packaging and images with different purposes and findings. Torrico et al. [8] used the system and biosensory app to assess consumers’ physiological and emotional responses when tasting different chocolate samples and image stimuli and compared the responses from Asian and Western consumers, finding differences between both cultures. Another study was conducted using image stimuli from the GAPED database with the earliest version of the integrated system and biosensory app and it was found that, by using subconscious responses along with six conscious questions, it is possible to cluster the images according to the GAPED classification [22]. A study using thermochromic labels as stimuli included the use of the integrated system along with eye-tracking techniques to assess acceptability from consumers, finding that there is no difference in preference and acceptability when assessing physical labels compared to their virtual version [23]. Gonzalez Viejo et al. [3] used the system along with an electroencephalogram device to assess acceptability of different beers and were able to develop machine-learning models using only the biometric responses as inputs to predict the level of liking (low and high) of some sensory attributes such as flavor, carbonation, and overall liking with over 80% accuracy. Likewise, a study including eye-tracking techniques was conducted to assess the acceptability and preference of different beer samples through their visual evaluation by watching videos from their pouring and they were able to develop a machine-learning model using the biometric responses from consumers, along with the objective parameters related to foam and color, maeasured using a robotic pourer RoboBEER, as inputs to classify the samples into high and low liking of the foamability with 82% accuracy [7]. The biosensory app without the video and IRTI recording has also been used in other beer studies to assess the intensity of the samples’ descriptors using a 15 cm nonstructured scale based on the quantitative descriptive analysis method obtaining the expected results according to the samples tested [24,25].

This integrated system and biosensory app have the advantage of being mobile and that they can be used not only in a sensory laboratory, but also in any environment, which only needs to have a WiFi connection. Despite that, there are other software such as Compusense (Compusense Inc., Guelph, ON, Canada) and RedJade sensory software (Curion, Redwood City, CA, USA) that have the ability of being mobile, but they can only be used to present sensory forms for self-reported responses and does not have the capability of recording biometrics. This feature of gathering self-reported responses and nonintrusive biometrics (heart rate, blood pressure, facial expressions, among others) in an integrated manner and remotely is uninque and, therefore, along with the development of machine-learning models, has a wide range of potential uses in the assessment of food, beverages, packaging, and any other nonfood products.

## 5. Conclusions

The newly developed integrated camera system and biosensory app have shown to be an accurate and convenient tool that, at the same time, reduces the time of data gatheriang and analysis. Furthermore, the use of nonintrusive methods to obtain data from unconscious responses is a more reliable way to assess these responses without affecting participant behavior due to stress caused by contact with, or exposure to, several sensors. Data obtained using the biosensory app and integrated cameras can be readily used to create machine-learning models to further assess other products based on biometrics and/or physiological responses without the necessity of asking questions to panelists. There is considerable potential in this sense in the application of these machine-learning models on consumers that do not have the skills to follow questionaires based on different scales, such as children and the elderly with mental ilnness such as dementia.

## Figures and Tables

**Table 1:** Example of the output of data obtained from the biosensory app in text file when opened in Excel.

Assessment	Image	Sample	Baby	Face scale	Progress	85	Time: 10:23:03
Assessment	Image	Sample	Dark room	Face scale	Progress	13	Time: 10:24:48
Assessment	Image	Sample	Spider	Face scale	Progress	22	Time: 10:26:06
Assessment	Image	Sample	Stairs	Face scale	Progress	48	Time: 10:28:55
Assessment	Image	Sample	Boat	Face scale	Progress	79	Time: 10:29:23
Assessment	Image	Sample	Dentist	Face scale	Progress	30	Time: 10:31:01
Assessment	Image	Sample	Door	Face scale	Progress	52	Time: 10:31:53
Assessment	Image	Sample	Wheel	Face scale	Progress	55	Time: 10:32:14
Assessment	Image	Sample	Dog	Face scale	Progress	94	Time: 10:33:23
Rank	Sample Baby	Position 1	Sample Dog	Position 2	Sample Boat	Position 3	
